# Does it take two to tango?

**DOI:** 10.1007/s12471-023-01846-4

**Published:** 2024-01-05

**Authors:** Daniel Mol, Erik A. Stel, Irene E. Hof

**Affiliations:** Department of Cardiology, OLVG Hospital, Amsterdam, The Netherlands

## Answer

**Fig. 1:**
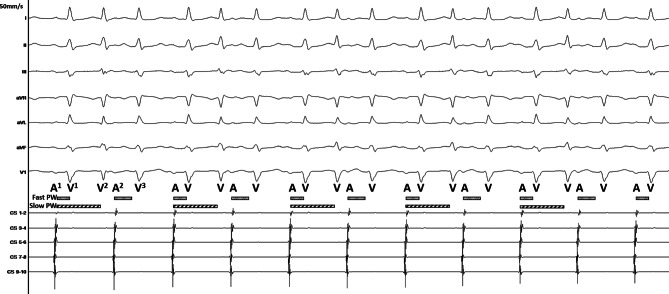
Intracardiac tracing. For a detailed explanation, please see the text. *Fast PW* fast pathway, *Slow PW* slow pathway, *A* atrial activation, *V* ventricular activation

**Fig. 2:**
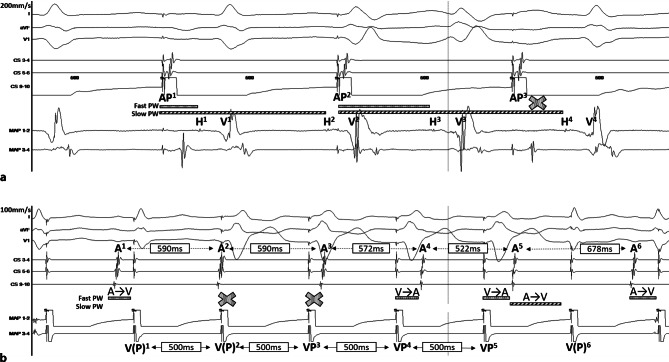
Intracardiac tracing during atrial (**a**) and ventricular pacing (**b**). For a detailed explanation, please see the text. *AP* atrial pacing, *H* His bundle potential, *V* ventricular activation, *A* atrial activation, *VP* ventricular pacing, *V(P)* (pseudo) fusion, *Fast PW* fast pathway, *Slow PW* slow pathway, *A→V* conduction from the atrium to the ventricle, *V→A* conduction from the ventricle to the atrium. Coronary sinus diagnostic catheter (*CS*), ablation catheter (*MAP*) located on the His bundle (**a**) and in the right ventricle (**b**)

The intracardiac tracing presented in Fig. 1 shows that one atrial beat (A^1^) is followed by two QRS complexes (V^1^ and V^2^) and that the second atrial beat (A^2^) produces only one QRS complex (V^3^). This pattern is repetitive along the tracing. We were able to reproduce this rhythm by atrial pacing (AP) (Fig. 2a) and observed that each V was preceded by a His bundle potential (H), suggesting that the rhythm is supraventricular. Ventricular pacing (VP) at a cycle length of 500 ms repeatedly resulted in a VA Wenckebach: the atrial activation sequence changes (A^4^ and A^5^), the A–A interval decreases (A^3^–A^4^ and A^4^–A^5^), and the VA interval lengthens (VP^4^–A^4^, VP^5^–A^5^) (Fig. 2b). Notably, a narrow QRS complex [V(P)^6^] followed after A^5^. As the activation wavefront was conducted retrogradely from VP^5^ through the fast pathway to the atrium (A^5^), the fast pathway must be refractory. Therefore, the narrow QRS complex has to result from a second AV pathway, which is, in this case, a slow pathway (Fig. 2b; [1]). Thus, Fig. 1 shows a non-reentrant AV-nodal tachycardia (dual AV-nodal response) resulting from anterograde fast and slow pathway conduction [2]. The extremely prolonged slow pathway conduction (A^1^–V^2^) enables the His bundle to conduct the activation wavefront into a second QRS complex. Then, after A^2^, the slow pathway is refractory, and only the fast pathway conducts the activation wavefront (V^3^). In Fig. 2a, the same phenomenon occurred: AP^1^ initiates H^1^ and H^2^. We cannot conclude if H^4^ is a result of slow pathway conduction after AP^2^ or fast pathway conduction after AP^3^. The AP^3^–H^4^ time is shorter than the AP^2^–H^3^ time. Therefore, we assume that this beat also conducts through the slow pathway. However, the AP^2^–H^3^ time might be prolonged because of conduction in the relative refractory period. We successfully eliminated the non-reentrant AV-nodal tachycardia via slow pathway modification.

## References

[1] Veenhuyzen GD, Quinn FR, Wilton SB, Clegg R, Mitchell LB (2011). Diagnostic pacing maneuvers for supraventricular tachycardia: part 1. Pacing Clin Electrophysiol Pace.

[2] Wu D, Denes P, Dhingra R, Pietras RJ, Rosen KM (1975). New manifestations of dual A-V nodal pathways. Eur J Cardiol.

